# High-Performance On-Chip Silicon Beamsplitter Based on Subwavelength Metamaterials for Enhanced Fabrication Tolerance

**DOI:** 10.3390/nano11051304

**Published:** 2021-05-14

**Authors:** Raquel Fernández de Cabo, David González-Andrade, Pavel Cheben, Aitor V. Velasco

**Affiliations:** 1 Instituto de Óptica Daza de Valdés, Consejo Superior de Investigaciones Científicas (CSIC), 28006 Madrid, Spain; david.gonzalez@csic.es (D.G.-A.); a.villafranca@csic.es (A.V.V.); 2 National Research Council Canada, Ottawa, ON K1A 0R6, Canada; pavel.cheben@nrc-cnrc.gc.ca

**Keywords:** photonic integrated circuits, silicon photonics, power division, beamsplitter, Y-junction, subwavelength metamaterial, ultra-broadband, fabrication-tolerant

## Abstract

Efficient power splitting is a fundamental functionality in silicon photonic integrated circuits, but state-of-the-art power-division architectures are hampered by limited operational bandwidth, high sensitivity to fabrication errors or large footprints. In particular, traditional Y-junction power splitters suffer from fundamental mode losses due to limited fabrication resolution near the junction tip. In order to circumvent this limitation, we propose a new type of high-performance Y-junction power splitter that incorporates subwavelength metamaterials. Full three-dimensional simulations show a fundamental mode excess loss below 0.1 dB in an ultra-broad bandwidth of 300 nm (1400–1700 nm) when optimized for a fabrication resolution of 50 nm, and under 0.3 dB in a 350 nm extended bandwidth (1350–1700 nm) for a 100 nm resolution. Moreover, analysis of fabrication tolerances shows robust operation for the fundamental mode to etching errors up to ±20 nm. A proof-of-concept device provides an initial validation of its operation principle, showing experimental excess losses lower than 0.2 dB in a 195 nm bandwidth for the best-case resolution scenario (i.e., 50 nm).

## 1. Introduction

The silicon-on-insulator (SOI) integrated photonic platform has been successfully exploited in a wide variety of fields, from telecom and datacom systems [1,2] to biochemical sensors [3], LIDAR systems [4], microspectrometers [5,6,7] and supercontinuum generation [8], among many others. The expansion into these diverse application fields has been made possible by the inherent benefits of the SOI platform, including large capacity of integration due to high refractive-index contrast [9] and low-cost mass production provided by the compatibility with complementary metal oxide semiconductor (CMOS) fabrication processes [10]. Conversely, the strong modal confinement results in SOI devices with high sensitivity to geometrical deviations from nominal design. This constraint is also present in power splitting components, a fundamental functionality in most silicon photonic integrated circuits [11] and, specifically, in an extensive range of applications including wavelength- and mode-division multiplexing [12], optical phased arrays [13] and on-chip spectrometers [6].

State-of-the-art power division structures such as directional couplers, multimode interference (MMI) devices or power splitters based on slot and adiabatic waveguides entail shortcomings in terms of reduced operational bandwidth, high sensitivity to fabrication deviations or large footprints [14,15,16,17,18,19,20,21]. Directional couplers [14], despite a significant improvement in their manufacturing tolerances through geometrical optimization [15] and phase control sections [16], still present comparatively limited operational bandwidth. While bent directional couplers [17] have achieved a broadband response, they are affected by a strong sensitivity to manufacturing deviations. Slot waveguides [18] and adiabatic couplers [19] have demonstrated good performance over a wide bandwidth; however, these devices have considerably larger footprints. Similarly, MMI couplers offer numerous advantages, such as relatively small size and relaxed manufacturing tolerances [20], but their bandwidth is limited by the strong modal dispersion in multimode SOI waveguides. Different structures have also been proposed for power splitting, including inverse tapers [21], adiabatic tapers [22] and photonic crystals [23], which are limited by their narrow bandwidth. 

Symmetric Y-junctions, consisting of a stem waveguide which branches into two diverging arms, are one of the most widely used power splitters and belong to the very roots of integrated optics. Y-junction studies were first conducted in the 1970s [24,25], with the first cascaded 1 × 8 power splitters being presented on ion-exchanged glass in the 1980s [26]. Nowadays, Y-junctions are routinely incorporated, for example, in ultra-high speed, high-quality lithium niobate electro-optic modulators [27]. Due to the simplicity of its design and operation principle [28,29], we consider these devices to be of special interest for SOI platforms, particularly for applications involving cascaded power splitting (e.g., integrated microspectrometers [30]. Moreover, Y-junctions with a bimodal stem waveguide offer a strong potential in datacom applications of growing interest, such as mode division multiplexing [12,31,32]. The transition between the stem and arms is nearly lossless and wavelength independent for small enough branching angles and a perfectly sharp junction tip between said branches [28]. However, the latter condition is hindered in real scenarios by the finite resolution of fabrication processes, hence requiring the application of more complex structures and optimization algorithms, such as slotted Y-junctions [18] or particle swarm optimization (PSO) [33]. This is particularly stringent in deep-ultraviolet (UV) lithography [34], with a substantially larger minimum feature size (MFS) compared to electron-beam (e-beam) technology. Moreover, since the junction tip is located at the midpoint of the stem waveguide, coinciding with the fundamental mode power maximum, deviations from the tip nominal design particularly penalize losses for the fundamental mode. Conversely, first-order modes present a zero-power profile at their central point, enabling power lobe separation without significant losses.

Subwavelength grating (SWG) metamaterials, since their first demonstration in silicon waveguides [35,36,37,38,39,40], have been advantageously used as a powerful tool for overcoming performance limitations of conventional silicon-based integrated photonic devices [41,42]. SWGs are periodic arrangements of different dielectric materials with a grating period (Λ) substantially smaller than the wavelength (*λ*) of the propagating light [43]. Under this condition, the medium acts on a macroscopic level as a homogeneous metamaterial which combines the optical properties of its dielectric constituents (e.g., effective index, dispersion, anisotropy), hence enabling the customization of the medium optical response through geometrical design. This innovative solution has been successfully applied to fiber-chip couplers, on-chip polarization management, mode-division multiplexing and integrated interferometer arrays, to name a few examples [6,12,44]. Specifically, subwavelength metamaterials have been applied to different power splitting architectures such as directional couplers [45,46,47] or MMIs [48,49], providing compact devices with enhanced performance over a broad bandwidth [50].

In this work, we incorporate an SWG metamaterial in a symmetric Y-junction to effectively reduce mode confinement around the junction tip and, hence, mitigate fundamental mode loss penalty caused by MFS limitations. Two different resolution scenarios have been taken into account for the optimization of the SWG Y-junction: (i) with an MFS of 100 nm, corresponding to current deep-UV fabrication process (dry lithography) and (ii) with an MFS of 50 nm for emerging high-resolution processes in photonic foundries (immersion lithography). Full three-dimensional finite-difference time-domain (3D FDTD) simulations show negligible fundamental mode excess losses (EL) within an ultra-broad bandwidth in excess of 300 nm for both MFS scenarios. In addition, our device presents robust fabrications tolerances to over- and under-etching deviations of up to ±20 nm. A proof-of-concept device was fabricated, showing experimental excess losses lower than 0.2 dB in a 195 nm bandwidth for the best-case resolution scenario.

## 2. Principle of Operation and Device Design

**Figure 1:**
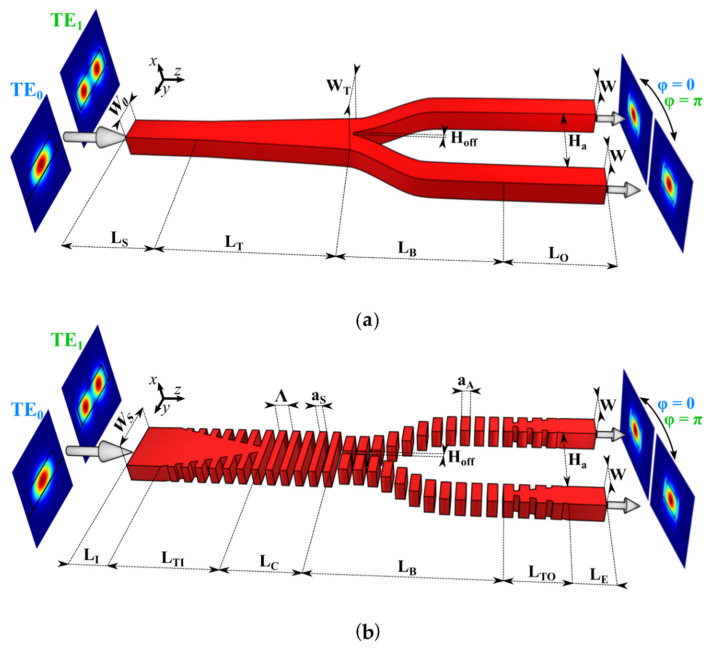
Schematic of (**a**) a conventional symmetric Y-junction and (**b**) SWG Y-junction. These two devices operate for both TE_0_ and TE_1_ modes.

As a reference framework for our proposed device, let us first consider a conventional symmetric Y-junction as depicted in Figure 1a, operating for fundamental and first-order transverse electric (TE) modes. The device comprises an input multimode waveguide (stem) of width *W*_0_ and length *L_s_*, and two single-mode S-shaped output arms of width *W* = *W*_0_/2, length *L_B_* and final separation *H_a_*, followed by output straight waveguides of length *L_O_*. S-shaped waveguides are typically used to implement lateral displacements connecting two parallel straight waveguides due to their reduced losses [51]. An adiabatic taper of length *L_T_* is also included to adapt the stem waveguide to the width of both arms at the fork (*W*_T_ = 2*W* + *H_off_*). When the divergence angle at the junction between the two arms is small enough to consider an adiabatic transition [28], the input fundamental TE mode (TE_0_) injected at the stem is divided into two in-phase TE_0_ modes at the output arms due to the symmetry of the device. Similarly, when the first-order TE mode (TE_1_) is injected, the power is again equally divided into two TE_0_ modes of equal amplitude at the output arms, but with a π phase difference (φ) between them. In order to account for the MFS constraint of the fabrication process, we consider a gap of width *H_off_* between the two arms at the junction tip.

Our proposed device, displayed in Figure 1b, operates analogously to a conventional symmetric Y-junction, but incorporates SWG metamaterials in both input and output waveguides, while preserving the same arm offset (*H_off_*). Arm width (*W*) and final separation (*H_a_*) are maintained identical as in the conventional Y-splitter for the sake of comparison. The input strip waveguide of length *L_I_* and width *W_S_* evolves into an SWG waveguide of length *L_C_* through an adiabatic taper (length *L_TI_*). This SWG region is key to reduce the modal confinement of the TE_0_ mode and subsequently to minimize the radiation loss at the fork and improve its excess losses. Furthermore, the use of subwavelength structures allows to define geometrical parameters (period, duty cycle and *H_off_*) with larger values than the considered MFS (i.e., 50 nm and 100 nm). In order to minimize mode mismatch at the interface between the input stem and the output arms, we utilized different duty cycles on both sides, *DC_S_* = *a_S_*/Λ and *DC_A_* = *a_A_*/Λ, where *a_S_* and *a_A_* are the length of the silicon segments in the stem and in the arms, respectively, considering a constant period Λ.

The device was optimized for an SOI platform with a core waveguide thickness of 220 nm and both top and buried silicon dioxide layers. At a wavelength of 1550 nm, the material refractive indices were *n_Si_* ∼ 3.48 and *n_SiO_*_2_ ∼ 1.44. The device was simulated using a 3D FDTD solver [52] for two different fabrication resolution limits: 50 nm and 100 nm. Therefore, the parameter *H_off_* was modified accordingly to each MFS scenario. The width of the Y-junction arms was *W* = 500 nm, ensuring compatibility with conventional interconnection waveguides. An SWG period of Λ = 220 nm was selected to avoid radiation and Bragg regimes. The list of the remaining geometrical design parameters is provided in Table 1.

**Figure 2:**
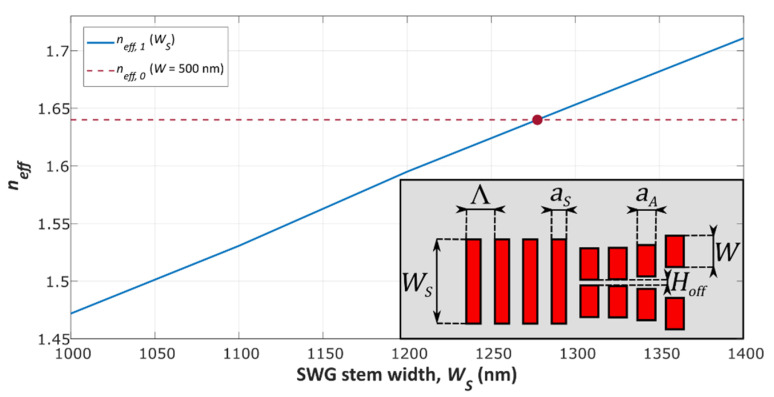
Effective index of the Bloch–Floquet TE_1_ mode for different widths of the SWG stem waveguide. The effective index of the Bloch–Floquet TE_0_ mode in a 500 nm wide SWG waveguide is represented with a dashed red line.

The width of the SWG stem waveguide was optimized to avoid a weak confinement of the Bloch–Floquet TE_1_ mode, which would lead to high TE_1_ excess losses (*EL_TE_*_1_) due to substrate leakage or mode radiation. TE_1_ mode splitting can be enhanced by selecting a wider SWG waveguide width, at the expense of a stronger confinement for the Bloch–Floquet TE_0_ mode and, therefore, higher TE_0_ excess losses (*EL_TE_*_0_). Figure 2 shows the effective index of the Bloch-Floquet TE_1_ mode (*n_eff_*_,1_) supported by the SWG stem waveguide as a function of the waveguide width. The effective index of the Bloch–Floquet TE_0_ mode (*n_eff_*_,0_) supported by the arms is also shown with a dashed red line. On this account, a width of the SWG stem waveguide of *W_S_* = 1200 nm was chosen as a compromise between *EL_TE_*_0_ and *EL_TE_*_1_.

**Figure 3:**
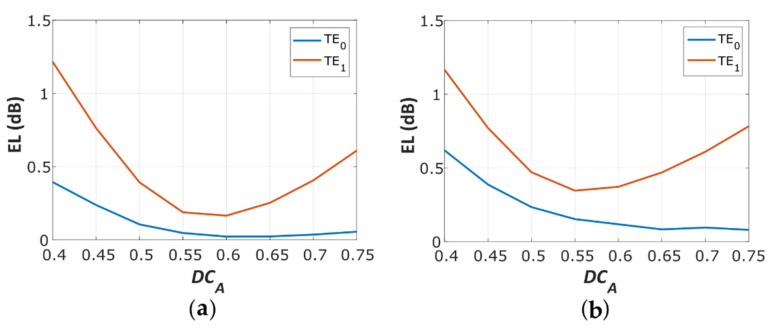
Calculated excess loss of the SWG Y-junction for variable *DC_A_* (*DC_S_* = 50%), for (**a**) MFS = 50 nm; (**b**) MFS = 100 nm.

To further optimize mode matching at the stem-arms interface, we judiciously adjusted the duty cycle on both parts of the device. For this purpose, we swept EL for different *DC_A_* while keeping a constant *DC_S_* of 50% (see Figure 3). We assumed two additional restrictions, i.e., that the chosen *DC_A_* cannot violate the MFS and that the optimum *DC_A_* values *EL_TE_*_0_ and *EL_TE_*_1_ may not necessarily be identical. For the MFS of 50 nm, the optimal loss balance for both TE_0_ and TE_1_ modes was achieved with a *DC_A_* = 60% (see Figure 3a). For the MFS of 100 nm, we found minimum EL for TE_1_ at *DC_A_* = 55% (see Figure 3b). 

## 3. Simulation Results and Tolerance Analysis

**Figure 4:**
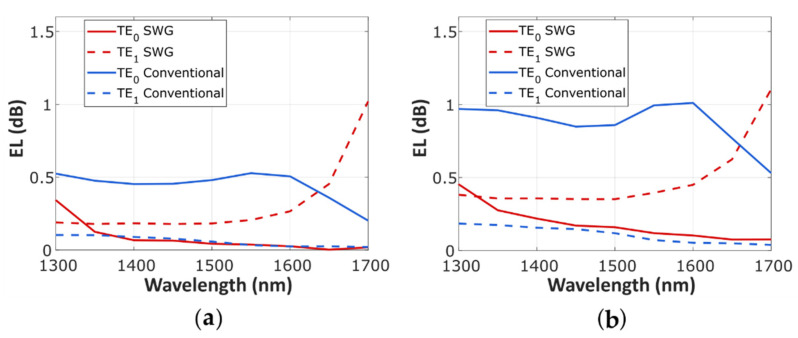
Calculated TE_0_ (solid curve) and TE_1_ (dashed curve) excess loss of the SWG Y-junction (red) compared to the conventional Y-junction (blue): (**a**) MFS = 50 nm, optimized SWG Y-junction with *DC_A_* = 60%; (**b**) MFS = 100 nm, optimized SWG Y-junction with *DC_A_* = 55%.

The performance comparison between the optimized SWG Y-junction (red) and its conventional counterpart (blue) is shown in Figure 4 (*EL_TE_*_0_ solid curve, *EL_TE_*_1_ dashed curve). For an MFS of 50 nm (Figure 4a), our device shows an excellent performance in a broad bandwidth of 300 nm, with *EL_TE_*_0_ below 0.1 dB for a wavelength range from 1400 nm to 1700 nm, and under 0.3 dB for the TE_1_ mode in a 1300–1600 nm window. For comparison, *EL_TE_*_0_ is reduced by 0.35 dB compared to the conventional splitter in a 250 nm bandwidth (1350–1600 nm), while *EL_TE_*_1_ is only slightly increased. Considering the MFS of 100 nm (Figure 4b), the SWG Y-junction exhibits *EL_TE_*_0_ as low as 0.3 dB in a 350 nm bandwidth (1350–1700 nm) and *EL_TE_*_1_ under 0.45 dB in a 300 nm wavelength range (1300–1600 nm). A significant reduction for *EL_TE_*_0_ is achieved at the central design wavelength (1550 nm), from 0.99 dB for a conventional Y-junction down to 0.12 dB for the SWG Y-junction. Despite a minor increase in *EL_TE_*_1_, the sum of both EL values is significantly reduced for the SWG device compared to the conventional Y-junction, providing a more even performance for both modes, for both 100 nm and 50 nm MFS designs. This outstanding performance is achieved in a broad bandwidth of 300 nm (1300–1600 nm), with the *EL_TE_*_0_ + *EL_TE_*_1_ under 0.5 dB being the figure of merit. The SWG Y-junction shows improved performance for both 100 nm and 50 nm MFS designs, while the performance of the conventional Y-junction degrades rapidly with increasing MFS. Simulations also confirmed that the effect of temperature changes on device performance were negligible for variations of ±10 K for both modes (TE_0_ or TE_1_), as well as for the two MFS designs.

**Figure 5:**
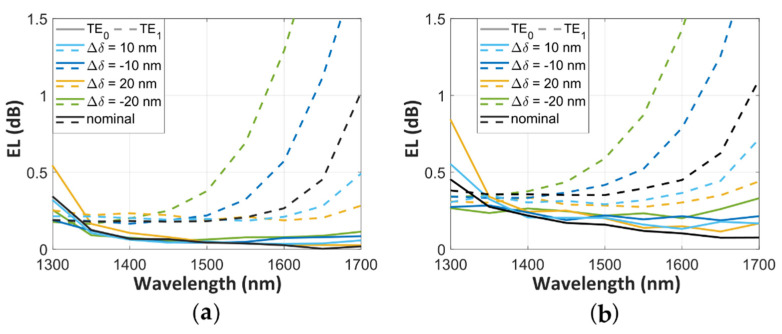
Tolerances to fabrication errors of Δ*δ* = ± 10, ± 20 nm for TE_0_ (solid curves) and TE_1_ (dashed curves) for the SWG Y-junction with (**a**) MFS = 50 nm and (**b**) MFS = 100 nm.

We also evaluated the fabrication tolerance of the SWG Y-junction to etching errors of Δ*δ* = ± 10 nm and Δ*δ* = ± 20 nm from our nominal design, as illustrated in Figure 5. For this purpose, we resized the whole device by adding to the length and width of the silicon segments the corresponding deviation, since we consider fabrication errors as absolute variations of the waveguide dimensions. Then, *a_S_*′ = *a_S_ +* Δ*δ* and *a_A_*′ *= a_A_ +* Δ*δ* are the lengths of the silicon segments at the stem and the arms of the SWG Y-junction, and the width at the stem and of the arms are *W_S_*′ *= W_S_ + Δδ* and *W_A_*′ *= W +* Δ*δ*, respectively. For both MFS values, i.e., 50 nm (Figure 5a) and 100 nm (Figure 5b), the SWG Y-junction performance degradation is observed predominantly for the TE_1_ mode when Δ*δ* is negative, i.e., for over-etching errors. By contrast, the TE_0_ mode exhibits robust tolerances, particularly for the MFS = 50 nm.

## 4. Fabrication and Experimental Characterization

**Figure 6:**
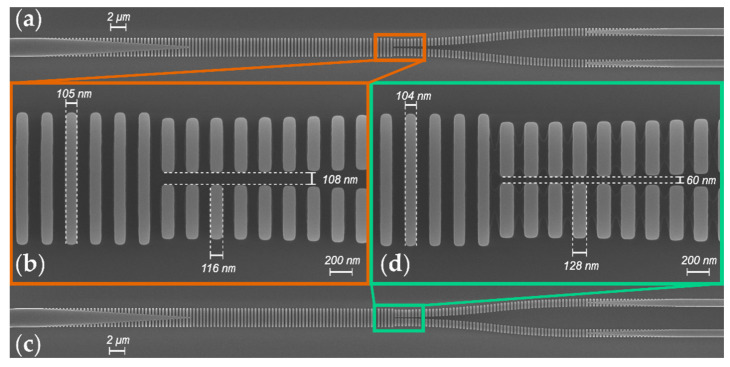
Scanning electron microscope images, for an MFS = 100 nm of (**a**) the complete SWG Y-junction and (**b**) the fork. For an MFS = 50 nm, SEM pictures of (**c**) the complete device and (**d**) the fork.

A proof-of-concept device was fabricated using SOI wafers with the 220 nm thick Si layer and 2 µm thick buried oxide (BOX). E-beam lithography was used to define the pattern, and the 220 nm thick Si layer was fully etched by inductively coupled plasma reactive ion etching. A SiO_2_ upper cladding was deposited via chemical vapor deposition to protect the devices. Figure 6 shows the scanning electron microscope (SEM) images of the fabricated devices prior the cladding deposition. SEM image of the subwavelength Y-junction with an MFS of 100 nm is presented in Figure 6a, with a more detailed view of the tip in Figure 6b. Adhering to this previous arrangement, SEM images of the splitter with an MFS of 50 nm can be seen in Figure 6c,d. Detailed SEM image analysis shows a slight over etching, with deviations below Δ*δ* < −10 nm in SWG segments of both arms and stem. A Mach-Zehnder interferometer (MZI) comprising two SWG Y-splitters was used to evaluate the coupler performance. High-efficiency and broadband SWG edge couplers [53,54] were used to couple the light in and out of the chip within the entire operational bandwidth of the device.

**Figure 7:**
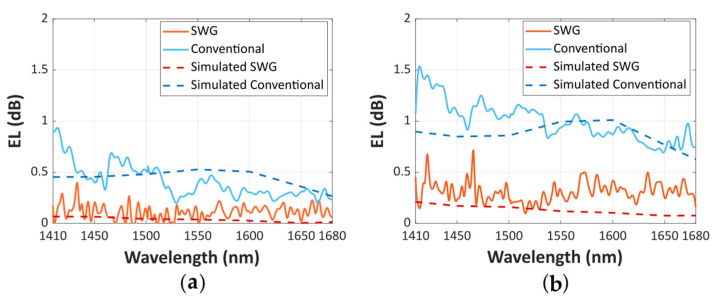
Measured EL for the TE_0_ mode for the SWG Y-junction (red) and the conventional Y-junction (blue) when (**a**) MFS = 50 nm and (**b**) MFS = 100 nm. Simulation results are also included for reference in all cases (dashed).

The fabricated device was characterized with two tunable lasers sweeping the wavelength range from 1.41 to 1.68 µm, fully covering the S, C, L and U telecom bands, and partially the E-band. Polarization at the chip input was controlled through a three-paddle fiber polarizer followed by a linear polarizer, a half-wave plate and a lensed polarization maintaining fiber. The polarization state was verified using a free-space polarimeter for the entire wavelength range (1.41–1.68 µm). The polarization state at the chip output was monitored with a Glan-Thompson polarizer, and a 40× microscope objective was used to focus the light onto a germanium photodetector. The difference between the transmittance of the measured MZI transmittance maxima and a reference waveguide, with the same waveguide length and number of bends as the MZI structure, allowed us to estimate the excess loss due to the SWG Y-junction. In order to conduct a conservative evaluation on the performance of our device, we chose the reference waveguide with the lowest measured losses among those available. Two reference Y-junctions (with an MFS of 100 nm and 50 nm, respectively) were also characterized in the 1410–1680 nm range to compare the performance of the SWG Y-junction with that of the conventional counterpart. The measured loss *EL_TE_*_0_ is shown in Figure 7.

The fabricated SWG Y-junction shows *EL_TE_*_0_ under 0.72 dB over the full bandwidth of 270 nm (1410–1680 nm) for the MFS = 100 nm. This value is reduced below 0.5 dB in a 210 nm bandwidth (1470–1680 nm). For the MFS = 50 nm, the *EL_TE_*_0_ is further reduced under 0.4 dB for the entire measured wavelength range, and under 0.22 dB in a 195 nm bandwidth (1485–1680 nm). This experimental performance implies significant improvement compared to the reference conventional Y-junction for both MFS scenarios, and particularly for the higher MFS case. Conventional Y-junctions show higher EL in all analyzed ranges, and a greater deterioration for shorter wavelengths, demonstrating the potential of SWG for circumventing fabrication resolution limitations. Table 2 summarizes the main parameters of our SWG Y-junction, compared with the performance of the state-of-the-art power splitters.

## 5. Discussion and Conclusions

We have proposed a new type of high-performance power splitter based on a Y-junction that incorporates subwavelength metamaterials. This strategy substantially reduces fundamental mode losses arising from limited fabrication resolution, particularly near the junction tip. For a high-resolution scenario (MFS = 50 nm), simulated excess losses for the fundamental mode are below 0.1 dB in an ultra-broad bandwidth of 300 nm (1400–1700 nm), and under 0.3 dB for the first-order mode in a 1300–1600 nm window. Considering a 100 nm MFS, our design presents EL for both TE_0_ and TE_1_ modes below 0.5 dB in 300 nm bandwidth (1300–1600 nm). Compared with a conventional Y-junction, this yields a reduction in TE_0_ loss at the central design wavelength (1550 nm) from 0.99 dB down to 0.12 dB, with only a small penalty on TE_1_ loss, the latter indeed being irrelevant for single-mode operation. 

Furthermore, our device demonstrates robust fabrication tolerances to etching errors up to ±20 nm, particularly for the TE_0_ mode. Our simulation results have been validated by a proof-of-concept experimental device, yielding and EL < 0.22 dB in a 195 nm bandwidth (1485–1680 nm) for an MFS = 50 nm and EL < 0.5 dB in a 210 nm bandwidth (1470–1680 nm) for an MFS = 100 nm. Additional experimental characterization of the device, including TE_1_ measurements and cascaded stages for enhanced accuracy, is expected in future works.

We believe that the SWG metamaterial engineered Y-junction power splitter will be useful for a wide range of applications of silicon photonic integrated circuits, with promising prospects for mode-division multiplexing, sensing, spectroscopy and any other application in which beam splitters are a cornerstone for optical power distribution.

## Figures and Tables

**Table 1:** Conventional and SWG Y-junction geometrical parameters.

Design	Parameter	Symbol	Value (μm)
SWG and conventional Y-junctions	Arm width Arm final separation Arm length	*W* *H_a_* *L_B_*	0.5 1.5 12.3
Conventional Y-junction	Stem waveguide length Taper length Output section length	*L_S_* *L_T_* *L_O_*	13 4 9
SWG Y-junction	Input strip width Input strip length Input SWG taper Output SWG taper Central SWG section Output strip length	*W_S_* *L_I_* *L_TI_* *L_TO_* *L_C_* *L_E_*	1.2 2 10 6 13 3

**Table 2:** Experimental performance comparison of state-of-the art power splitters. (* Values estimated from manuscript figures and data).

Ref	Structure	Bandwidth (nm)	*EL_TE_*_0_ (dB)	MFS (nm)	Length (μm)
[16]	Directional coupler	88	<1.0	200	31.4
[17]	Bent directional coupler	80	<1.0	110	50
[18]	Slotted Y-junction	390	<1.0	100	200
[20]	MMI coupler	60	<1.0	500	27
[21]	Inverse tapers	40	<4.0 *	100	16.1
[22]	Adiabatic tapers	100	<0.6	200	40
[29]	Tapered Y-junction	100	<0.3	0	30
[33]	PSO Y-junction	80	<1.0	200	2
[47]	SWG directional coupler	65	<1.0	90	4.5
[45]	SWG directional coupler	200	<1.0	110 *	17.3
[49]	SWG MMI	325	<1.0	95 *	25.4
This work	SWG Y-junction	270	<0.4	50	41.3
This work	SWG Y-junction	270	<0.7	100	41.3

## Data Availability

The data presented in this study are available on request from the corresponding author.
